# Genetic and Antigenic Diversity of *Neisseria meningitidis* Serogroup B Strains in Vietnam

**DOI:** 10.3390/pathogens14050487

**Published:** 2025-05-15

**Authors:** Trieu Phi Long, Vo Viet Cuong, Bui Thi Lan Anh, Trinh Van Toan, Vu Thi Loan, Pham Viet Hung, Le Thi Lan Anh, Nguyen Ngoc Tan, Luong Thi Mo, Le Van Khanh, Hoang Van Tong

**Affiliations:** 1 Graduate University of Science and Technology, Vietnam Academy of Science and Technology, Hanoi 100000, Vietnam; 2 Department of Microbiology, Military Institute of Preventive Medicine, Hanoi 100000, Vietnam; 3 Institute of Tropical Medicine, Joint Vietnam-Russia Tropical Science and Technology Research Center, Hanoi 100000, Vietnam; 4 Southern Branch of the Joint Vietnam-Russia Tropical Science and Technology Research Center, Ho Chi Minh City 700000, Vietnam; 5 Institute of Biomedicine and Pharmacy, Vietnam Military Medical University, Hanoi 100000, Vietnam; 6 Department of Pathophysiology, Vietnam Military Medical University, Hanoi 100000, Vietnam; 7 Vietnamese-German Center for Medical Research (VG-CARE), Hanoi 100000, Vietnam

**Keywords:** meningitis, *N. meningitidis*, genetic diversity, antigenic variation, Vietnam

## Abstract

Background: *Neisseria meningitidis* (*N. meningitidis*) is a leading cause of acute meningitis and is classified into 13 serogroups, six of which are predominantly associated with invasive meningococcal disease. This study aimed to investigate the genotype, subgenotype, and antigenic profiles of *N. meningitidis* serogroup B strains isolated in Vietnam. Methods: Genotyping was performed on 106 *N. meningitidis* strains isolated from clinical samples from Vietnamese patients and nasopharyngeal *swabs* of healthy adolescents between 2019 and 2024. The genetic profiles, including the porA, porB, fetA, fHbp, abcZ, adk, aroE, fumC, gdh, pdhC, and pgm genes, were analyzed using Sanger sequencing and bioinformatic methods. Results: We found that 84.9% of the strains carried VR3 families 36 or 35-1, with VR1, VR2, and VR3 families 22-25, 14, and 36 being the most prevalent. Among the 106 serogroup B isolates, 20 variants of the porB allele 3 were identified, with porB 3-1212 being the most frequent (30.2%). Dominant PorB variable loops included L1.6, L4.5, L5.7, L6.6, and L7.13. fHbp variant group 2 was predominant (104/106 strains), and 12 FetA allele variants were identified, with F1-7 being the most common (47.2%). Three clonal complexes were identified, and clonal complex ST-32 was the most predominant. Fifty-five strains (51.9%) belonged to sequence types that have not yet been assigned to any clonal complexes, and 15 strains (14.1%) with allelic profiles were not assigned to STs. The 3-253 and 3-1212 alleles of porB, the F1-7 variant of FetA, the ST-44 and ST-1576 sequence types, and the ST-41/44 complex were observed more frequently in patients compared to asymptomatic carriers, suggesting their association with more virulence. Conclusions: This study showed a high genetic and antigenic diversity of *N. meningitidis* serogroup B isolates in Vietnam, with VR3 family 36 most common and porB 3-1212 as the predominant allele. fHbp variant group 2 and FetA allele F1-7 were most frequent. ST-32 was the dominant clonal complex, though many strains remained unassigned, highlighting the need for ongoing molecular surveillance.

## 1. Introduction

Meningitis is an infection of the tissues surrounding the brain and spinal cord, caused by various bacteria, viruses, fungi, and parasites. Outbreaks of meningitis are common in the ‘Meningitis Belt’, a region spanning 26 countries in Sub-Saharan Africa. Bacterial meningitis is the most severe form, responsible for approximately 250,000 deaths annually worldwide [1]. According to the World Health Organization (WHO), *Neisseria meningitidis* (*N. meningitidis*) is one of the four primary bacteria that cause acute meningitis, with incidence rates of up to 1000 cases per 100,000 population [1]. Globally, approximately 236,000 deaths and 2.51 million incident cases of meningitis, with *N. meningitidis* accounting for about 13.6% of deaths, were estimated [2]. The incidence rate of invasive meningococcal disease in Vietnam was estimated to be 0.02 per 100,000 population and 7.4 in children under 5 years of age [3].

*N. meningitidis*, also known as meningococcus, is an aerobic, gram-negative encapsulated bacterium that resides in the human pharynx. When the immune system is weakened or the pharynx is inflamed, the bacteria can breach the mucosal barrier, enter the bloodstream, and cause hemorrhagic skin lesions or, in severe cases, purpura fulminans. In the brain, meningococci can invade the meninges, leading to meningitis [4]. These severe forms of the disease have a mortality rate of up to 50% if left untreated [5]. Vaccination remains the most effective strategy for preventing outbreaks and reducing the burden of this disease.

The antigenic structure of *N. meningitidis* includes capsular polysaccharides, lipooligosaccharides (LOS), and outer membrane proteins (OMPs) like *PorA*, *PorB*, *fHbp*, and *FetA*. Capsular polysaccharides are crucial for developing polysaccharide and conjugate vaccines [6]. *N. meningitidis* is classified into 13 serogroups based on the polysaccharide capsule (A, B, C, D, 29-E, H, I, K, L, W-135, X, Y, and Z), with six (A, B, C, W-135, X, and Y) responsible for most invasive meningococcal disease [7]. Conjugate vaccines effectively target serogroups A, C, W, and Y. However, the structural similarity of MenB capsular polysaccharides to human neuronal molecules limits immunogenicity, complicating vaccine development against serogroup B [8]. Although the MenACWY vaccine is available in Vietnam for adolescents and adults, the four-component meningococcal B vaccine (4CMenB) has recently been recommended for routine vaccinations in Vietnam for infants [9].

Serogroup B currently accounts for the highest percentage of invasive meningococcal disease cases worldwide, making the development of a broadly protective MenB vaccine essential [6,10]. Meningococcal Outer Membrane Vesicles (OMVs) are key targets for vaccine development, with examples like VA-MENGOCOC-BC, MenBvac, and MeNZB used against group B meningococcal disease in Cuba, Norway, and New Zealand, respectively [11]. The major OMPs, critical components of OMV vaccines, are divided into five classes: class 1 (PorA), class 2/class 3 (PorB), class 4 (Rmp), and class 5 (Opa and Opc) [12]. *N. meningitidis* is classified into subtypes based on PorA and serotypes based on PorB epitopes. PorA, a cation-selective transmembrane protein with two variable regions (VR1 and VR2), induces antibacterial antibodies and is a major target for recombinant MenB vaccines [13,14]. However, high variability in VR1 and VR2 reduces the vaccine’s specificity across different strains. The VR3 region, with lower variability, is considered a promising candidate for vaccine development, though its immunogenic potential remains unclear [15,16]. PorB, an anion-selective porin, is subdivided into allelic variants PorB2 and PorB3. It has been shown to elicit immune responses, including antibody production in mice and functional immune responses in infant sera post-vaccination [17].

FetA (Ferric Enterobactin Receptor A), an iron-regulated outer membrane protein, is another component of several meningococcal OMV vaccines. FetA is immunogenic due to its antigenic variation region, which is accessible to antibodies above the outer membrane [18]. Like PorA, the efficacy of FetA-based vaccines is limited by antigenic variability. However, combining PorA and FetA variants has been shown to improve vaccine coverage against invasive meningococcal disease in Europe [19,20,21]. Factor H binding protein (FHbp), a lipoprotein on the meningococcal surface that binds human factor H, is classified into three variants (var1, var2, and var3) or two subfamilies (A and B, corresponding to variants 2/3 and 1, respectively) [22]. FHbp is included in both licensed protein-based MenB vaccines, MenB-FHbp and MenB-4C. Unlike FetA and PorA, recombinant FHbp vaccines offer broad immunity against serogroup B meningococcal disease, as the generated antibodies exhibit cross-immunity within the same subfamily [23].

Understanding bacterial population structure is crucial for developing and implementing region-specific vaccine policies. Sequencing techniques for antigen-encoding genes such as fHbp, fetA, and the variable regions of porA and porB, along with multilocus sequence typing (MLST), are widely used to classify and track the distribution of antigenic variants. Numerous new meningococcal sequence types (STs) have been identified and added to the database. This study aimed to investigate the genotype, subgenotype, and *porA*, *porB*, *fetA*, and *fHbp* profiles of *N. meningitidis* serogroup B strains isolated in Vietnam.

## 2. Materials and Methods

### 2.1. Ethical Statement

Written informed consent was obtained from all study participants. This study has been considered and approved by the Joint Vietnam-Russia Tropical Science and Technology Research Center (approval ref: 1047/CN-HĐĐĐ).

### 2.2. Selection of N. meningitidis Isolates

A total of 106 *N. meningitidis* strains of serogroup B were in our study, including 25 strains that were cultured from cerebrospinal fluid and blood of patients and 81 isolates cultured from nasopharyngeal *swabs* of healthy adolescents in Vietnam between 2019 and 2024 (Table 1). All samples were confirmed as *N. meningitidis* through culture isolation and polymerase chain reaction (PCR) testing of cerebrospinal fluid, blood, or petechial lesions following the routine instructions. Patients who tested negative for *N. meningitidis* or declined to participate in the study were excluded.

### 2.3. N. meningitidis Isolation and Confirmation

The gold standard for diagnosing meningococcal infection is the isolation of *N. meningitidis* from sterile body fluids, such as cerebrospinal fluid (CSF) and blood (collected before using antibiotics). Blood samples were cultured at a 1:10 ratio in Brain Heart Infusion (BHI) medium supplemented with 5% Fildes enrichment, then incubated at 37 °C under 10% CO_2_. Nasopharyngeal specimens were cultured on chocolate agar supplemented with a combination of vancomycin, colistin, and nystatin (VCN antibiotics) and incubated at 35–37 °C under 3–5% CO_2_ with humidity maintained at ≥50%. CSF samples were preserved in a trans-isolate transport medium or cultured immediately on 5% blood agar or chocolate agar and promptly transported to the laboratory. Bacteria were identified by Gram stain and using biochemical tests: oxidase reaction and API^®^ NH cards (bioMérieux, Lyon, France). Pure isolates were stored at −80 °C in brain heart broth medium with 15% glycerol.

### 2.4. Amplification and Sequencing

The *porA*, *porB*, *fetA*, *fHbp*, and seven housekeeping genes (*abcZ*, *adk*, *aroE*, *fumC*, *gdh*, *pdhC*, *pgm*) of *N. meningitidis* were amplified by PCR. The primer sequences for amplifying the genes are listed in the supplementary Tables S1 and S2. PCR reaction components and thermal conditions for amplification of study genes were described previously [24,25,26]. The PCR products were purified using the EZ-10 Spin Column Kit (Biobasic, Toronto, ON, Canada) and subsequently subjected to Sanger sequencing using the BigDye Terminator v3.1 Cycle Sequencing Kit on the ABI Prism 3100 Sequencer (Applied Biosystems, Carlsbad, CA, USA). The sequences of primers used in sequencing are presented in Table S3.

### 2.5. Bioinformatic and Statistical Analyses

The DNA sequences of *N. meningitidis* strains were analyzed using the BioEdit program (https://bioedit.software.informer.com/7.2/ (accessed on 12 May 2025)) and aligned with CLUSTAL_X and BLAST (https://blast.ncbi.nlm.nih.gov/Blast.cgi (accessed on 12 May 2025)). Protein sequences were translated from DNA using the ExPASy translation tool (http://web.expasy.org/translate (accessed on 12 May 2025)). Genetic polymorphisms within the species were assessed using the multilocus sequence typing (MLST) method. The genotypes of seven housekeeping genes—*abcZ* (putative ABC transporter); *adk* (adenylate kinase); *aroE* (shikimate dehydrogenase); *fumC* (fumarate hydratase); *gdh* (glucose-6-phosphate dehydrogenase); *pdhC* (pyruvate dehydrogenase subunit); and *pgm* (phosphoglucomutase)—as well as the variable loop of *porB*; the VR1; VR2; and VR3 regions of *porA*; *fetA*; and *fHbp*; were classified using the PubMLST database (https://pubmlst.org/organisms/neisseria-spp (accessed on 12 May 2025)).

The frequencies were presented as percentages and tested using the chi-square or Fisher’s exact test, while quantitative variables were presented as medians and compared using the *t*-test or Mann–Whitney test. Odds ratios (OR) and 95% confidence intervals (CI) were calculated to assess the association of molecular characteristics with the virulence of *N. meningitidis*. All statistical analyses were performed using SPSS 25 software with a significance level set at a *p*-value of <0.05.

## 3. Results

### 3.1. Distribution of PorA Genotype

We found 23 different combinations of three families, VR1, VR2, and VR3, in this study, including 11 VR1 families, 19 VR2, and 5 VR3 families. The most common VR1 family was P1.22-25 (n = 34, 32.1%), followed by P1.7-2 (n = 21, 19.8%), P1.22 (n = 21, 19.8%), and P1.12-14 (n = 18, 16.2%). For the VR2 family, the most prevalent variants were 14 (n = 50, 47.2%), 13-20 (n = 18, 16.7%), and 13-9 (n = 14, 13.2%). We observed that 84.9% of the analyzed strains carried one of two VR3 family 36 (n = 49, 46.2%) and VR3 family 35-1 (n = 41, 38.7%). The three most common profiles were P1.22-25,14,36 (n = 28, 26.4%), P1.22,14,36 (n = 19, 17.9%), and P1.12-14,13-20,35-1 (n = 18, 17.0%). These three profiles represent 60.4% of the strains analyzed (Table 2).

### 3.2. Distribution of PorB Genotype and Its Association with the Virulence of N. meningitidis

Among the 106 meningococcal isolates analyzed, 20 variants of the *porB* allele 3 were identified within the serogroup B isolates. The porB 3-1212 allele was the most frequent (n = 32, 30.2%), followed by porB 3-980 (n = 19, 17.9%) and porB 3-922 (n = 18, 17.0%). We also examined the distribution of PorB variable loops and found that L1.6, L4.5, L5.7, L6.6, and L7.13 were the dominant loops (Table 3). These findings suggest that the *porB* allele 3-1212 may have a higher adaptive capacity or greater transmission efficiency compared to the other alleles. We compared *PorB* allele frequencies between strains isolated from patients and asymptomatic carriers; the 3-253 and 3-1212 alleles were observed more frequently in *N. meningitidis* strains isolated from patients compared to those in *N. meningitidis* isolates from asymptomatic carriers (OR = 4.9, 95% CI = 1.2–19.5, *p* = 0.006; OR = 3.2, 95% CI = 1.1–8.8, *p* = 0.011; respectively) (Table 4).

### 3.3. Distribution of fHbp Genotype in the N. meningitidis Strains

FHbp variant group 2 (subfamily A) was most common (n = 104, 98.1%), followed by group 1 (subfamily B) (n = 2, 1.9%). A total of 5 variants of subfamily A were found. The A22 variant was the most frequent (n = 62, 58.5%), followed by A32 (n = 26, 24.5%), A20 (n = 8, 7.5%), and A07 (n = 5, 4.7%). There were 1.9% of the strains that showed FHbp variants, 1 like the FHbp variant in the 4CmenB vaccine (Table 5). The majority of the identified strains had amino acid sequences corresponding to one of the two modules, III and VI. These findings suggest that the strong adaptive capacity of fHbp VG2 strains, carrying motifs III and VI, may play a key role in *N. meningitidis’* ability to evade the immune system and cause disease. We compared the frequencies of the Novartis variant group, subfamily, and module group between strains isolated from patients and asymptomatic carriers; however, the difference was not statistically significant (*p* > 0.05) (Table S4).

### 3.4. Distribution of FetA Genotype and Its Association with the Virulence of N. meningitidis

We identified 12 FetA allele variants, grouped into five families (F1, F2, F3, F4, and F5). The most common genotype was F1-7, found in 50/106 strains (47.2%), followed by F5-1 with 22/106 (20.8%) and F3-16 with 17/106 (16.0%). Together, these alleles accounted for 84.0% (89/106) of the total samples, indicating their clear dominance in the population. We also found that the frequency of the F1-7 genotype was significantly higher in patients compared to those in asymptomatic carriers, indicating that this genotype was associated with increased virulence in *N. meningitidis* (OR = 5.9, 95% CI = 2.1–18.3, *p* = 0.0001) (Table 6).

### 3.5. Distribution of MLST and Its Association with the Virulence of N. meningitidis

The results of Multilocus Sequence Typing (MLST) analysis of 106 *N. meningitidis* strains, based on combinations of seven housekeeping genes (*abcZ*, *adk*, *aroE*, *fumC*, *gdh*, *pdhC*, *pgm*), are shown in Table 7. A total of 10 different STs were identified among the 90 isolates. The most prevalent ST was the ST-1576 lineage, which was found in 36 strains. Seven of the STs, representing 34.9% (36/106) of the isolates, could be grouped into three clonal complexes. The identified clonal complexes included ST-32 (n = 19, 17.9%), ST-41/45 (n = 15, 14.1%), and ST-175 (n = 2, 1.9%). Fifty-five strains (51.9%) belonged to sequence types that have not yet been assigned to any clonal complexes by the *N. meningitidis* MLST website (http://pubmlst.org/neisseria (accessed on 12 May 2025)). Notably, 15 strains (14.1%) with allelic profiles were not assigned to STs.

We compared the frequencies of sequence type (ST) and clonal complex (CC) of *N. meningitidis* strains isolated from patients and asymptomatic carriers. The results showed that the frequencies of *ST-44* and *ST-1576* sequence types were significantly higher in *N. meningitidis* strains isolated from patients compared to those in *N. meningitidis* isolates from asymptomatic carriers (OR = 7.2, 95% CI = 1.8–30.7, *p* = 0.002; OR = 3.0, 95% CI = 1.1–8.1, *p* = 0.011; respectively). In addition, the ST-41/44 complex was isolated more frequently in patients compared to asymptomatic carriers (OR = 7.0, 95% CI = 1.9–27.1, *p* = 0.0003) (Table 8).

## 4. Discussion

Invasive meningococcal disease (IMD), caused by *N. meningitidis*, remains a significant global public health issue, particularly affecting infants and young children. Our findings regarding the characterization of PorB, FetA, and fHbp variants, as well as the identification of dominant clonal complexes in Vietnam, suggest valuable insights for vaccine design, molecular diagnostics, and epidemiological surveillance. The higher frequency of porB alleles 3-253 and 3-1212, FetA F1-7, and specific sequence types (ST-44, ST-1576, and the ST-41/44 complex) in clinical isolates compared to asymptomatic carriers suggests their association with increased virulence. This supports their potential use as molecular markers for risk assessment and the development of more targeted preventive measures, particularly during outbreaks.

Due to its initial mild and nonspecific symptoms, the disease can progress rapidly, resulting in a high mortality risk ranging from 4% to 20% within 48 h [9]. In Vietnam, the incidence of meningococcal disease in 2018 was reported at 0.02 per 100,000 population [3]. A prospective surveillance study conducted from 2000 to 2002 estimated the incidence rate of IMD among children aged 7–11 months to be 29.1 per 100,000 population. In response, Vietnam has recently launched a vaccination program with the four-component protein-based meningococcal B vaccine (4CMenB; Bexsero, GSK, London, UK), aiming to protect over 1 million infants over the next five years.

Serogroup B strains are prevalent in many countries, accounting for 30–40% of cases in the United States and up to 80% in Europe [6]. Our study found that 96.7% of the strains analyzed were serogroup B. According to surveillance data from 2012 to 2021, serogroup B IMD accounted for 82% of cases in Vietnam, while serogroup C accounted for 18% [9]. A study has conducted molecular characterization of *N. meningitidis* isolates in Vietnam; however, the analysis was constrained by a limited number of isolates [27]. Since 23 February 2024, the 4CMenB vaccine, which targets serogroup B meningococcal disease, has been approved for use in Vietnam. Developed using reverse vaccinology technology, this vaccine includes three recombinant antigens—factor H-binding protein (fHbp); Neisseria heparin-binding antigen (NhbA); and Neisseria adhesin A (NadA)—along with outer-membrane vesicles containing Porin A subtype P1.4 from the NZ98/254 strain [28]. Therefore, monitoring the circulation and evolution of serogroup B strains is crucial for evaluating the vaccine’s effectiveness.

Currently, serogroup B vaccines primarily use OMPs, particularly PorA and fHbp, as key antigens. PorA is one of the most highly expressed outer membrane proteins of *N. meningitidis* and has a strong ability to stimulate immune responses [29]. In our study, the majority of the strains (84.9%) carried one of two VR3 families, 36 or 35-1. In contrast, we identified 12 VR1 variants and 18 VR2 variants in these strains. These findings align with a study in Brazil, where 87.1% of the strains analyzed had one of the two VR3 variants (35 or 36), with 8 VR1 and 12 VR2 families also identified [16]. This supports the idea that the genetic stability of VR3 is greater than that of VR1 and VR2, possibly because PorA VR3 is less exposed to the extracellular environment, allowing it to evade the host immune system more effectively. We propose that VR3 families 36 and 35-1 may serve as promising candidates for developing next-generation vaccines against *N. meningitidis* serogroup B. Overall, P1.22-25,14,36, P1.22,14,36, and P1.12-14,13-20,35-1 were the predominant subtypes in our study. VR2 family 4, a component of the 4CMenB vaccine, was absent from strains. Results indicated low coverage of the PorA antigen component in the 4CMenB vaccine to *N. meningitidis* serogroup B isolates in Vietnam.

Besides porin A subgroup P1.4, fHbp (variant 1.1) is also one of the four major antigenic components of the 4CMenB vaccine [30]. FHbp is a key surface protein of *N. meningitidis* that helps the bacteria evade destruction by the innate immune system. In our study, the majority of strains were classified as fHbp VG2, with amino acid sequences corresponding to modules III and VI. These findings suggest that *N. meningitidis* strains with fHbp VG2 variants in motifs III and VI may have a stronger binding affinity for host Factor H, allowing them to evade the immune system more effectively and potentially enhancing their transmission efficiency. The 4CMenB vaccine uses fHbp variant 1.1 (Module I and VG1) as its primary antigen, which may result in reduced protective efficacy in Vietnam, where VG2 predominates. Although the vaccine offers good coverage and cross-immunity, we recommend regular monitoring of both VG1 and VG2 variants to update and optimize vaccine components.

PorB is a transmembrane protein that functions as a channel, allowing small molecules to pass between the bacterial cell and its environment, thereby contributing to intracellular balance [31]. Although PorB is not the main antigen in vaccines against *N. meningitidis* serogroup B, it is believed to induce functional immune responses in infant sera following vaccination [17]. In our study, the most common PorB alleles were 3-1212 (30.2%), followed by 3-980 (17.9%) and 3-922 (17.0%) out of the twenty identified. Additionally, the variable loops L1.6, L4.5, L5.7, L6.6, and L7.13, which correspond to these alleles, were dominant in the population. This may be due to structural changes in the variable loops of PorB, allowing these alleles to evade antibodies or enhance host cell invasion efficiency. However, PorB has not yet been a primary antigen in currently licensed vaccines, which have focused on more conserved antigens such as fHbp, NadA, and NhbA. In addition, our results suggested that the *N. meningitidis* strains with 3-253 and 3-1212 alleles had more virulence compared to those carrying other alleles.

Due to the diversity of *PorA* variants, current OMV vaccines that rely on PorA as the primary antigen require the identification of additional candidates, such as fetA, to ensure broad coverage of various strains [7]. FetA is a surface-expressed protein in *N. meningitidis* that plays a crucial role in iron acquisition from the external environment, supporting bacterial growth. We identified 12 FetA allele variants, with F1-7, F5-1, and F4-6 being the most common, accounting for 84.0% (89/106) of the total strains. Similarly, a study in Brazil (2016–2018) identified 17 FetA allele variants, with F5-1 being one of the most prevalent [32]. Another study found the FetA allele variant F5-1 to be common in Western Australia between 2000 and 2011 [23]. Given its widespread prevalence, we suggest that FetA allele F1-7, F5-1 could be a promising candidate for future vaccine development. Of 12 identified FetA allele variants, the F1-7 variant was associated with an increased virulence of *N. meningitidis* strains. This could be explained by the contribution of the F1-7 variant of FetA to more efficient attachment and invasion of the bacteria to the host cells.

Finally, we employed MLST to monitor the genetic diversity and classify *N. meningitidis* strains in Vietnam. The observed clonal complexes included ST-32 (19 strains), ST-41/44 (15 strains), and ST-175 (2 strains). The ST-32 and ST-41/44 complexes are hypervirulent and commonly associated with serogroup B IMD [33]. In Vietnam, ST-1576 has predominated for over four decades. ST-1576 and variants ST-13860, ST-12962, ST-11005, ST-11006, and ST-11013 were the main causes of IMDs and carriage, representing more than 56%. The significant prevalence of the ST-1576 lineage, which is strongly associated with chloramphenicol resistance [34]. Similarly, the ST-44 and ST-1576 sequence types and ST-41/44 complex were observed more frequently in patients compared to asymptomatic carriers, suggesting that these sequence types and clonal complex were associated with a higher virulence.

In conclusion, our study provides a detailed analysis with high genetic and antigenic diversity of *N. meningitidis* serogroup B in Vietnam, with VR3 family 36 most common and porB 3-1212 as the predominant allele. fHbp variant group 2 and FetA allele F1-7 were most frequent. ST-32 was the dominant clonal complex, though many strains remained unassigned. The porB alleles 3-253 and 3-1212, FetA F1-7, and specific sequence types (ST-44, ST-1576, and the ST-41/44 complex) were associated with increased virulence. These findings underscore the need for ongoing monitoring and evaluation of the pathogenic potential of these strains and the effectiveness of current serogroup B meningococcal vaccines in Vietnam.

## Figures and Tables

**Table 1:** Main characteristics of the *N. meningitidis* isolates.

Characteristics	All Isolates	From Patients	From Asymptomatic Carriers	*p* Value
**Number of Isolates;** n (%)	106 (100)	25 (23.6)	81 (76.4)	NA
**Age;** median (min–max)	19 (1–22)	19 (1–22)	19 (18–21)	NS
**Sex:** male/female	98/8	19/6	79/2	0.004
**Clinical diagnosis**	106 (100)			<0.0001
*Meningitis*	25 (23.6)	25 (23.6)	0	
*Asymptomatic Carrier*	81 (76.4)	0	81 (76.4)	
**Treatment outcome**				<0.0001
*No treatment, n (%)*	81 (76.4)	0	81 (76.4)	
*Recovery, n (%)*	23 (21.7)	23 (21.7)	0	
*Death, n (%)*	2 (1.9)	2 (1.9)	0	
**Location of Isolation**				<0.0001
*North; n (%)*	66 (62.3)	16 (15.1)	50 (47.2)	
*Central, n (%)*	11 (10.4)	9 (8.5)	2 (1.9)	
*South, n (%)*	29 (27.4)	0	29 (27.4)	

NA: not applicable; *p*-values were calculated by Fisher’s exact test.

**Table 2:** The PorA subgenotypes of *N. meningitidis*.

No. of Isolates (n = 106)	Genosubtype		
	VR1	VR2	VR3
19	22	14	36
2	22	9	35-1
18	12-14	13-20	35-1
1	18	25	35-1
1	19	15	36
1	19	25	38-1
1	20	2	36-2
1	21-2	28	36-2
1	21-2	ND	36-2
3	22-11	15-25	36
28	22-25	14	36
4	22-25	14	38-1
2	22-25	14-80	36
1	5-2	10-1	36-2
1	7	16-103	35
14	7-2	13-9	35-1
2	7-2	13-1	35-1
1	7-2	13-1	38-1
1	7-2	13-2	35-1
1	7-2	13-15	35-1
1	7-2	13-18	35-1
1	7-2	4-18	36
1	ND	2-48	38-1

ND: Non-determined.

**Table 3:** The distribution of *PorB* genotypes of *N. meningitidis*.

No. of Isolates (n = 106)	PorB Loop					PorB Allele
	*L1*	*L4*	*L5*	*L6*	*L7*	
17	6	7	ND	10	ND	3-980
1	ND	7	ND	10	ND	3-980
1	6	7	ND	ND	ND	3-980
13	7	7	10	12	11	3-922
5	7	7	10	12	ND	3-922
31	6	5	7	6	13	3-1212
1	6	7	11	ND	13	3-1212
2	7	12	10	12	11	3-901
13	6	7	11	11	5	3-253
1	7	7	10	12	11	3-974
3	3	7	20	10	9	3-913
1	9	7	15	6	7	3-48
1	6	7	ND	11	5	3-755
1	6	7	20	10	9	3-71
2	6	7	11	9	5	3-393
1	4	5	7	9	ND	3-254
3	9	7	13	9	12	3-860
1	6	7	ND	10	ND	3-1154
1	11	7	20	10	9	3-410
7	ND	5	11	11	5	3-368

ND: Non-determined.

**Table 4:** Association of *PorB* allele with the virulence of *N. meningitidis*.

*PorB* Allele	All Isolates	From Patients	From Asymptomatic Carriers	*p* Value	OR (95% CI)
*3-48*	1 (0.9)	0	1 (1.2)	NS	ND
*3-71*	1 (0.9)	1 (4)	0	NS	ND
***3-253***	**13 (12.3)**	**7 (28)**	**6 (7.4)**	**0.006**	**4.9 (1.2–19.5)**
*3-254*	1 (0.9)	0	1 (1.2)	NS	ND
*3-368*	7 (6.6)	0	7 (8.6)	NS	ND
*3-393*	2 (1.9)	0	2 (2.5)	NS	ND
*3-410*	1 (0.9)	1 (4)	0	NS	ND
*3-755*	1 (0.9)	1 (4)	0	NS	ND
*3-860*	3 (2.8)	0	3 (3.7)	NS	ND
*3-901*	1 (0.9)	0	1 (1.2)	NS	ND
*3-913*	3 (2.8)	0	3 (3.7)	NS	ND
*3-922*	19 (17.9)	4 (16)	15 (18.5)	NS	ND
*3-974*	1 (0.9)	0	1 (1.2)	NS	ND
*3-980*	19 (17.9)	0	19 (23.5)	NS	ND
*3-1154*	1 (0.9)	0	1 (1.2)	NS	ND
***3-1212***	**32 (30.2)**	**11 (44)**	**21 (25.9)**	**0.011**	**3.2 (1.1–8.8)**

ND: Non-determined. NS: not significant.

**Table 5:** The variants of *fHbp* of the *N. meningitidis* strains.

No. of Isolates (n = 106)	Novartis Variant Group	Sub Family	Module Group	Modular Variable Segment Allele				
				*A*	*B*	*C*	*D*	*E*
**62**	**2**	**A22**	VI	A1.1	B1.1	C2.2	D1.1	E2.1
8	2	A20	VI	A1.1	B1.1	C2.4	D1.1	E2.9
26	2	A32	III	A1.1	B1.1	C2.1	D2.1	E2.1
5	2	A07	III	A1.1	B1.1	C2.1	D2.1	E2.3
2	2	A24	VI	A1.1	B1.1	C2.12	D1.1	E2.4
2	1	B	ND	ND	B1.1	ND	D1.2	E1.53
1	2	ND	ND	A1.19	B2.1	C2.2	D1.1	E2.2

ND: Non-determined.

**Table 6:** The distribution of *FetA* genotype and its association with the virulence of *N. meningitidis*.

FetA_VR Genotypes	All Isolates	From Patients	From Asymptomatic Carriers	*p* Value	OR (95% CI)
*F1-2*	1 (0.9)	0	1 (1.2)	NS	ND
*F1-5*	2 (1.9)	0	2 (2.5)	NS	ND
***F1-7***	**50 (47.2)**	**18 (72)**	**32 (39.5)**	**0.0001**	**5.9 (2.1–18.3)**
*F1-145*	1 (0.9)	1 (4)	0	NS	ND
*F2-4*	1 (0.9)	0	1 (1.2)	NS	ND
*F3-3*	2 (1.9)	0	2 (2.5)	NS	ND
*F3-16*	17 (16)	4 (16)	13 (16)	NS	ND
*F4-6*	7 (6.6)	2 (8)	5 (6.2)	NS	ND
*F4-67*	1 (0.9)	0	1 (1.2)	NS	ND
*F5-1*	22 (20.8)	0	22 (27.2)	NS	ND
*F5-9*	1 (0.9)	0	1 (1.2)	NS	ND
*F5-64*	1 (0.9)	0	1 (1.2)	NS	ND

**Table 7:** Gene fragments used in MLST analysis.

No. of Isolates (n = 106)	Multilocus Sequence Typing (MLST)							Sequence Type	Clonal Complex
	abcZ	adk	aroE	fumC	gdh	pdhC	pgm		
1	4	5	2	9	9	11	9	New	
1	4	5	2	9	9	11	224	New	
14	4	5	2	9	9	11	17	13863	
1	4	5	2	9	9	11	523	New	
1	4	10	5	4	6	155	8	New	
1	4	274	2	1183	9	343	17	New	
1	140	5	9	56	921	34	165	New	
1	4	5	2	9	9	11	165	New	
17	4	10	5	4	6	55	8	230	CC32
1	350	2	6	25	24	6	21	New	
1	9	10	9	9	9	6	9	1009	CC41/44
2	2	10	2	17	9	11	20	New	
2	343	2	34	25	365	68	21	5542	
13	9	6	9	9	9	6	9	44	CC41/44
1	222	3	58	275	9	5	255	New	
36	140	5	9	173	175	34	165	1576	
2	4	10	5	4	6	55	8	32	CC32
1	9	92	9	9	9	6	9	new	
1	9	6	6	25	9	6	9	15346	CC41/44
1	6	7	4	25	26	247	8	new	
2	6	7	4	56	26	18	8	175	CC175
1	350	2	6	25	279	6	22	new	
1	4	5		53	26	41	165	new	
3	140	5	4	173	175	34	165	13074	

**Table 8:** The association of MLST with the virulence of N. meningitidis.

MLST	All Isolates	From Patients	From Asymptomatic Carriers	*p* Value	OR (95% CI)
**Sequence type**					
*ST-32*	2 (1.9)	1 (4)	1 (1.2)	NS	ND
***ST-44***	**13 (12.3)**	**8 (32)**	**5 (6.2)**	**0.002**	**7.2 (1.8–30.7)**
*ST-175*	2 (1.9)	0	2 (2.5)	NS	ND
*ST-230*	15 (14.2)	3 (12)	12 (14.8)	NS	ND
*ST-1009*	1 (0.9)	1 (4)	0	NS	ND
***ST-1576***	**37 (34.9)**	**12 (48)**	**25 (30.9)**	**0.01**	**3.0 (1.1–8.1)**
ST-5542	2 (1.9)	0	2 (2.5)	NS	ND
*ST-13074*	2 (1.9)	0	2 (2.5)	NS	ND
*ST-13863*	13 (12.3)	0	13 (16)	NS	ND
*ST-15346*	1 (0.9)	0	1 (1.2)	NS	ND
*New*	18 (17)	0	18 (22.2)	NS	ND
**Clonal complex**					
*ST-32 complex*	17 (16)	4 (16)	13 (16)	NS	ND
*ST-41/44 complex*	**15 (14.2)**	**9 (36)**	**6 (7.4)**	**0.0003**	**7.0 (1.9–27.1)**
*ST-175 complex*	2 (1.9)	0	2 (2.5)	NS	ND
*Not assigned*	72 (67.9)	12 (48)	60 (74.1)	0.015	ND

ND: Non-determined. NS: not significant

## Data Availability

The authors confirm that the data supporting the findings of this study are available within the article and are also available on request.

## Supplementary Materials

The following supporting information can be downloaded at: https://www.mdpi.com/article/10.3390/pathogens14050487/s1, Table S1: Primer sequences used for amplification of FetA, fHbp, PorA, and PorB; Table S2: Primer sequences used for MLST; Table S3: Primer sequences used for amplification and sequencing; Table S4: Association of fHbp genotype with the virulence of *N. meningitidis*.
