# Supplementary material for: Genetic and Antigenic Diversity of Neisseria meningitidis Serogroup B Strains in Vietnam
Source: Pathogens. 2025 May 15;14(5):487. doi: 10.3390/pathogens14050487 (PMC12114657; doi:10.3390/pathogens14050487)
Supplement: Supplementary file 1 [file pathogens-14-00487-s001.zip › pathogens-3593178-supplementary.pdf]

Supplementary table 1: Primer sequences used for amplification of FetA, fHbp, PorA and PorB

Primers		Sequence (5' - 3')[1]	Product length (bp)	Reference
fetA_Fw	Forward	CGGCGCAAGCGTATTCGG	1189	(1)
fetA_Rv	Reverse	CGCGCCCAATTCGTAACCGTG		
fHbp_Fw	Forward	GTCCGAACGGTAAATTATYGTG	895	(2)
fHbp_Rv	Reverse	CTATTCTGVGTATGACTAG		
PorA_Fw	Forward	AAACTTACCGCCCTCGTA	1100	(3)
PorA_Rv	Reverse	TTAGAATTTGTGGCGCAAACCGAC		
PorB2_Fw	Forward	GAAGTTTCTCGCGTAAAARAT	966	This study
PorB2_Rv	Reverse	CATGCTGGCAGTTTKTTC		
PorB3_Fw	Forward	GCGTAGAAACTTCCCGCTC	810	This study
PorB3_Rv	Reverse	CAGAAGTGCGTTTGG AGAAGT		

[1]IUPAC Nucleotide Designations: V = A, C or G; Y = C or T; R = A or G; K = G or T; Fw: Forward; Rv: Reverse

Supplementary table 2 : Primer sequences used for MLST

Primers		Sequence (5' - 3')	Product length (bp)	Reference
abcZ-Fw	Forward	TGTTCCGCTTCGACTGCCAAC	433	(2)
abcZ-Rv	Reverse	TCCCCGTCGTAAAAAACAATC		
adk-Fw	Forward	CCAAGCCGTGTAGAATCGTAAACC	465	(2)
adk-Rv	Reverse	TGCCCAATGCGCCCAATAC		
aroE-Fw	Forward	TTTGAAACAGGCGGTTGCGG	490	(2)
aroE-Rv	Reverse	CAGCGGTAATCCAGTGCGAC		
fumC-Fw	Forward	TCCCCGCCGTAAAAGCCCTG	465	(2)
fumC-Rv	Reverse	GCCCGTCAGCAAGCCCAAC		
gdh-Fw	Forward	CTGCCCCCGGGGTTTTCATCT	501	(2)
gdh-Rv	Reverse	TGTTGCGCGTTATTTCAAAGAAGG		
pdhC-Fw	Forward	CCGGCCGTACGACGCTGAAC	480	(2)
pdhC-Rv	Reverse	GATGTCGGAATGGGGCAAACA		
pgm-Fw	Forward	CTTCAAAGCCTACGACATCCG	450	(2)
pgm-Rv	Reverse	CGGATTGCTTTCGATGACGGC		

Fw: Forward; Rv: Reverse

Supplementary table 3: Primer sequences used for amplification and sequencing

Primers		Sequence (5' - 3') [1]	Reference
fetA S12	Forward	TTCAACTTCGACAGCCGCCTT	(1)
fetA S15	Reverse	TTGCAGCGCGTCRTACAGGCG	
fHbpP1	Forward	GTCCGAACGGTAAATTATYGTG	(2)
fHbpP2	Reverse	CTATTCTGVGTATGACTAG	
PorA_Fw	Forward	AAACTTACCGCCCTCGTA	This study
PorA_Rv	Reverse	TTAGAATTTGTGGCGCAAACCGAC	
PorB2_Fw	Forward	GAAGTTTCTCGCGTAAAARAT	This study
PorB2_Rv	Reverse	CATGCTGGCAGTTTKTTC	
PorB3_Fw	Forward	GCGTAGAAACTTCCCGCTC	This study
PorB3_Rv	Reverse	CAGAAGTGCGTTTGGAGAAGT	
abcZ-S1A	Forward	AATCGTTTATGTACCGCAGR	(2)
abcZ-S2	Reverse	GAGAACGAGCCGGGATAGGA	
adk-S1A	Forward	AGGCWGGCACGCCCTTGG	(2)
adk-S2	Reverse	CAATACTTCGGCTTTCACGG	
aroE-S1A	Forward	GCGGTCAAYACGCTGRTK	(2)
aroE-S2	Reverse	ATGATGTTGCCGTACACATA	(2)
fumC-S1	Forward	TCCGGCTTGCCGTTTGTCAG	(2)
fumC-S2	Reverse	TTGTAGGCGGTTTTGGCGAC	(2)
gdh-S3	Forward	CCTTGGCAAAGAAAGCCTGC	(2)
gdh-S4C	Reverse	RCGCACGGATTCATRYGG	(2)
pgm-S1	Forward	CGGCGATGCCGACCGCTTGG	(2)
pgm-S2A	Reverse	GGTGATGATTTCGGTYGCRCC	(2)
pdhC-S1	Forward	TCTACTACATCACCCTGATG	(2)
pdhC-S2	Reverse	ATCGGCTTTGATGCCGTATTT	(2)

[1]IUPAC Nucleotide Designations: V = A, C or G; Y = C or T; R = A or G; K = G or T

Supplementary table 4: Association of *fHbp* genotype with the virulence of *N. meningitidis*

fHbp genotype	All Isolates	From Patients	From Carriers	P value	OR (95%CI)
**fHbp Novartis Variant Group**				NS	ND
1	2 (1.9)	1 (4)	1 (1.2)		
2	104 (98.1)	24 (96)	80 (98.8)		
**fHbp Sub Family**				NS	ND
A	1 (0.9)	0	1 (1.2)		
A07	5 (4.7)	1 (4)	4 (4.9)		
A20	8 (7.5)	1 (4)	7 (8.6)		
A22	62 (58.5)	18 (72)	44 (54.3)		
A24	2 (1.9)	0	2 (2.5)		
A32	26 (24.5)	4 (16)	22 (27.2)		
B	2 (1.9)	1 (4)	1 (1.2)		
**fHbp Modul Group**				NS	ND
III	31 (29.2)	5 (20)	26 (32.1)		
IV	72 (67.9)	19 (76)	53 (65.4)		
ND	3 (2.8)	1 (4)	2 (2.5)		

References

1. Thompson EAL, Feavers IM, Maiden MCJ. Antigenic diversity of meningococcal enterobactin receptor FetA, a vaccine component. Microbiology (Reading). 2003;149(Pt 7):1849-58.
2. Organization WH. Laboratory Methods for the diagnosis of Meningitis caused by Neisseria meningitidis, Streptococcus pneumoniae, and Haemophilus influenzae. WHO MANUAL. 2011.
3. Taha MK, Alonso JM, Cafferkey M, Caugant DA, Clarke SC, Diggle MA, et al. Interlaboratory comparison of PCR-based identification and genogrouping of Neisseria meningitidis. J Clin Microbiol. 2005;43(1):144-9.
